# Contour of the Face

**Published:** 1847-10

**Authors:** J. Allen


*Extract from an Address delivered before the Mississippi Valley Association of Dental Surgeons, upon the Contour of the Face. By* DR. J. ALLEN. *August,* 1847.

MR. PRESIDENT:

In compliance with the duties assigned me at our last meeting, I will call your attention to the subject of the human face which in its most perfect state, presents a symmetrical rotundity that gives beauty and harmony to the form of the cheek, but which, by the ravages of time and disease, often become changed into a sunken, haggard or cadaverous form, changing so entirely the appearance of the lady or gentleman, that after an elapse of a few short years, distant friends on meeting them will scarce acknowledge a recognition.

The causes which produce this sad change in the face are generally the loss of the teeth, absorption of the alveolar processes, wasting of the flesh, &c., the two latter being consequent upon the former. That portion of the face which most usually falls in, is situated principally upon the exterior portion of the superior and inferior maxillary bones. The muscles which form this portion of the face are seven in number, viz: the zigomaticus major, zigomaticus minor, masseter, buccinnator or trumpetors muscle, levator labii, superioris, alaequi nasi, levator anguli oris, and orbicularis oris. When these muscles occupy their natural or proper position, they give a certain degree of rotundity to the cheek. This rotundity is sustained by the maxillary arches, alveolar processes, teeth, gums, &c.; but when these natural organs fail, the cheeks of many persons fall in, particularly those of a nervous sanguine temperament, and the bare replacing of the teeth in the usual mode, does *not* restore the form of the face to its original configuration; (I have reference more particularly to the side teeth;) hence the frequent objections that are urged against the removal of teeth, even when they are so far decayed as to render extraction necessary.

Having frequently encountered this haggard looking wreck of humanity in the face which seemed to bid defiance to our skill, we finally resolved to make an effort to surmount it. But without a compass or guide, without one ray of light from the pages of history to show that any thing of the kind had been previously accomplished in connexion with our profession, our efforts for a time, were not without their attendant difficulties, but ultimate success finally crowned our efforts.

Schooled in the art of contending with formidable difficulties, we have been taught to believe that whatever obstacles we meet with in our profession, MUST be overcome. The appendage by which the form of the face can be restored to its original contour, is made of gold; and connected with the teeth and surrounding parts in such a manner as to bring out the muscles that may have fallen in, and again sustain them in their proper position. In many cases this appendage may be quite small. If, for instance, the zigomaticus minor is the only muscle that has fallen in, it would require but little to restore it to its proper place; but if in addition to this, the zigomaticus major and masseter muscles had also fallen in, it would require a much larger structure to reproduce a symmetrical form. These are the muscles which most usually becomes sunken from the loss of the molar teeth.

The loss of the bicuspides, cuspidati and insicor teeth, cause a falling in of the buccinnator,—levator labii superioris alæqui nasi, levator angulis oris, and orbicularis oris.

[*To be continued.*]

